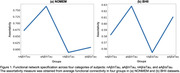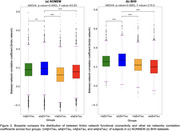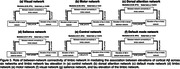# Remote associations between beta‐amyloid and tau are mediated by between‐network connectivity

**DOI:** 10.1002/alz.086460

**Published:** 2025-01-09

**Authors:** Seyed Hani Hojjati, Tracy A Butler, Mony J. de Leon, Ajay H Gupta, Siddharth Nayak, Jose A. Luchsinger, Gloria Chiang, Qolamreza R Razlighi

**Affiliations:** ^1^ Weill Cornell Medicine, New York, NY USA; ^2^ Weill Cornell Medicine, New York City, NY USA; ^3^ Brain Health Imaging Institute, Department of Radiology, Weill Cornell Medicine, New York City, NY USA; ^4^ Columbia University Medical Center, New York, NY USA

## Abstract

**Background:**

The accumulation of tau tangles and beta‐amyloid (Aβ) are hallmarks of Alzheimer's disease (AD). Despite the hypothesis that Aβ may trigger tau spread across remote brain regions, the specific pathological processes remain unclear.

**Methods:**

Our study utilized 18F‐Florbetaben Aβ positron emission tomography (PET), 18F‐MK6240 tau PET, and resting‐state functional magnetic resonance imaging (rs‐fMRI). We included 361 healthy control (HC) subjects from the Northern Manhattan Study of Metabolism and Mind (NOMEM) [mean age 64.96 ± 3.17 years, 230 females]. Among them, 46 subjects underwent follow‐up scans at 2 to 3 year intervals. Additionally, 120 HC subjects from the Brain Health Imaging Institute (BHII) studies were included (mean age 68.71 ± 6.16 years, 54 females). Cross‐sectional elderly subjects were categorized into four groups based on normal (n) and abnormal (a) levels of Aβ and tau, as compared to young normative subjects (age < 40): nAβ/nTau, aAβ/nTau, nAβ/aTau, and aAβ/aTau. Assortativity, a graph theory metric, was computed on the average functional connectivity maps for each group. We also compared the connectivity between the limbic network and other functional networks among these groups of subjects. Finally, using longitudinal subjects, we explored the role of between‐network connectivity of the limbic network in mediating the association between cortical Aβ in six functional networks and annual tau elevation in the limbic network.

**Results:**

Figure 1 illustrates that among the four groups in both datasets, aAβ/nTau subjects displayed the highest assortativity values (0.767 for NOMEM and 0.627 for BHII). In both datasets, aAβ/nTau showed significant increases (t‐value > 4.49, p‐value < 0.0001) in between‐network connectivity of the limbic network (Figure 2) compared with the other three groups. 14 Aβ‐positive subjects achieved highly significant results (p‐value < 0.013) for indirect relationships in all six functional networks (Figure 3).

**Conclusion:**

Our study highlights the early accumulation of Aβ and its role in increasing between‐network connectivity. We also demonstrated that between‐network connectivity mediates the remote relationships between Aβ and tau pathologies in the preclinical stages of AD.